# A survey of primary care patients’ readiness to engage in the de-adoption practices recommended by Choosing Wisely Canada

**DOI:** 10.1186/s13104-016-2103-6

**Published:** 2016-06-10

**Authors:** William Silverstein, Elliot Lass, Karen Born, Anne Morinville, Wendy Levinson, Cara Tannenbaum

**Affiliations:** Faculty of Medicine, Department of Medicine, University of Toronto, 30 Bond Street, Toronto, ON M5B 1W8 Canada; Centre de Recherche, Institut Universitaire de Gériatrie de Montréal, Université de Montréal, Montréal, QC Canada; Faculty of Medicine, Université de Montréal, Montréal, QC Canada; Faculties of Medicine and Pharmacy, Université de Montréal, Montréal, QC Canada

**Keywords:** Choosing Wisely, Patient educational materials, Health literacy, Shared-decision making

## Abstract

**Background:**

Strategies such as Choosing Wisely have been established to identify the overuse of interventions considered as low-value. Reduction of low-value practices will require patients to understand why certain interventions are no longer recommended. The objective of this study was to determine whether older adults accept the rationale for and perceive themselves ready to de-adopt annual electrocardiogram testing, imaging for low back pain, the use of antibiotics for sinusitis, the use of sedative-hypnotics for insomnia, and the use of antipsychotics to treat behavioural symptoms of dementia.

**Methods:**

A self-administered iPad survey was distributed to consecutive patients aged 50 years and older, presenting to three primary care outpatient practices in Ontario, Canada. Data from patients who were able and willing to complete the survey while waiting to see their physician were included. The survey queried knowledge, attitudes and behaviours around the targeted low-value interventions, before and after exposure to a Choosing Wisely Canada patient educational brochure on one of these five topics. A subset of patients agreed to participate in a semi-structured interview after their clinic visit.

**Results:**

Three-hundred and forty-four patients (mean age 63, range 50–88, 59 % female) read the materials and completed the survey. Forty-eight percent (95 % CI 43–53 %) intended to discuss the information with a healthcare provider. Forty-five percent (95 % CI 40–51 %) expressed a desire to change current low-value practices. Approximately two-thirds of those who indicated they would not change future behaviours explained that it was because they were already espousing the Choosing Wisely values. After reading the Choosing Wisely brochures, knowledge improved independent of age, sex and education in 48 % (95 % CI 38–57 %) of participants about electrocardiogram testing, in 74 % (95 % CI 65–82 %) about use of antipsychotics, in 66 % (95 % CI 52–78 %) about use of antibiotics for sinusitis, in 60 % (95 % CI 46–72 %) about imaging for low back pain, and in 40 % (95 % CI 26–55 %) about sedative-hypnotic use in the elderly.

**Conclusions:**

The majority of primary care patients seem ready to de-adopt low-value practices. Provision of education in clinic waiting rooms can help improve knowledge around unnecessary care.

## Background

Up to 30 % of all health care tests, treatments and procedures are estimated to be unnecessary [1–3]. Unnecessary care offers little to no clinical benefit, can harm patients, and wastes limited health care resources [4, 5]. The drivers of unnecessary care are multiple, complex, and contentious. Many physicians believe that patient demands and expectations account for the persistence of many low-value interventions [6, 7]. However emerging data suggest this is not always the case [8–10]. Evidence-informed patients can effectively drive discontinuation of low value prescriptions such as benzodiazepines in the elderly [11]. The strategy to support de-prescribing of medications is now being more broadly proposed to several classes of medications that may no longer be necessary, or where harm potentially outweighs benefit [12].

The Choosing Wisely initiative is now established in 18 countries around the world, including the USA, UK, Canada and Australia, to identify and reduce the overuse of interventions considered as low-value [13–15]. The International Choosing Wisely Top 10 list illustrates some examples of low-value care that have been universally accepted [15]. It is generally assumed that the onus lies on the physician to diminish rates of low-value interventions [16], but buy-in from patients is also required. To this end, Choosing Wisely patient educational brochures were developed to inform consumers why more care is not necessarily better [17].

The aim of this study was to determine whether primary care patients understand the rationale for and perceive themselves ready to de-adopt certain low-value interventions such as annual electrocardiogram testing, imaging for low back pain, the use of antibiotics for sinusitis, the use of sedative-hypnotics for insomnia, and the use of antipsychotics for patients with dementia. We also aimed to assess the impact of providing educational materials on patients’ perceptions towards low-value care practices.

## Methods

This is a cross-sectional survey of older adults aged 50 years and older, presenting to one of three primary care outpatient clinics in Ontario, Canada between July 2014 and March 2015. Potential participants who entered the waiting rooms of the clinics were systematically approached by trained research assistants to assess interest and eligibility in participating in a Choosing Wisely Canada (CWC) iPad survey while waiting for their appointment. Patients who never used an iPad were given an orientation by the research assistant. Exclusion criteria included not being fluent in English, inability to read due to visual impairment, and inability to comprehend due to cognitive impairment. We targeted patients aged 50 years and older, as the CWC topics chosen are most relevant to this age group.

Individuals who consented to participate in the survey were asked to choose one of five CWC topics on the iPad opening page. The five topics were strategically selected at the discretion of the research team from the initial CWC items available in April 2014 and thought to be relevant to all older adults in primary care. These topics were: annual electrocardiogram (ECG) testing, use of antipsychotic drugs for patients with dementia, use of antibiotics for treatment of sinusitis, imaging for low back pain, and sedative-hypnotic use in patients with insomnia. These five topics are frequently cited in the literature as common examples of unnecessary tests and treatments prescribed in primary care [1, 18–20]. After selecting a topic of interest, participants were asked to fill in a survey on the iPad, which included socio-demographic information and questions ascertaining knowledge and behaviours related to their topic of choice. The questions were modeled against the previously validated EMPOWER brochure [11, 23]. Participants were asked to indicate their level of agreement with statements in the brochure, on a 5-point Likert Scale comprised of “strongly agree/agree/unsure/disagree/strongly disagree”. Participants were then invited to read the CWC patient educational material on their topic of choice. Immediately after reading the CWC brochure, participants rescored the questions and indicated their intent to discuss the information with a health care provider, or change their behaviour with respect to the low-value intervention.

During the second half of the study, we invited all participants to engage in a structured interview after their primary care appointment, in order to better understand participants’ motivations for choosing one of the five CWC topics, and to gauge intent for discussing the CWC material or changing their behaviour. Participants who had time to stay and answer two open-ended questions were included. The questions were: “Why did you choose this particular brochure,” and “Will the information presented change your behaviour? If yes, how? If no, why not?”

### Statistics

Only data from participants who were able to complete both the pre-survey and post-survey prior to being called into their appointment were included. Descriptive statistics were used to characterize the participants. Proportions, with 95 % confidence intervals (CI), were used to estimate self-reported knowledge and behaviours for each CWC topic. Knowledge improvement pre- to post-exposure to the CWC educational materials was defined as a change from an incorrect to a correct answer about the topic after reading the brochure. A correct answer was defined as an endorsement of the response options “strongly agree or agree” or “strongly disagree or disagree”, as appropriate with statements about each topic. McNemar’s test for matched pairs analysis was used to examine changes within groups, stratified by each of the five topics, from baseline to post-intervention. Statistical significance for all analyses was set at p < 0.05 (two-sided tests). SPSS (SPSS Inc. Chicago, USA) was used for all analyses. Univariate logistic regression was used to ascertain whether demographic variables predicted knowledge improvement for each topic.

Responses to the structured interviews underwent thematic content analysis after data collection was complete. Themes were defined as a group of responses that described a unique phenomenon. Two members of the research team (WS, EL) separately coded the data into said themes, based on their interpretation. WS and EL then reconciled any differences by consensus building. Once each patient’s answers were categorized into themes, the number of responses allocated to each theme was calculated. The proportion of the number of times each theme recurred, over the total number of participants, was calculated. These analyses were conducted using Microsoft Excel (Microsoft Inc. Redmond, USA).

### Ethical issues and data confidentiality

The St. Michael’s Hospital, Trillium Health Partners and Women’s College Hospital Research Ethics Boards granted approval for the study in July 2014, December 2014 and December 2014, respectively. Consent to fill out the survey was all that was required to participate. All data was entered anonymously. At no point did patients reveal their name or contact information to the research assistant.

## Results

### Participation and characteristics of respondents

**Fig. 1:**
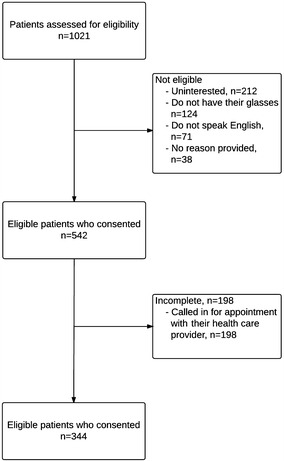
Flow of participants through the study

A total of 1021 participants were approached to participate. Just over half (n = 542, 53.1 %) consented. Three-hundred-and-forty-four participants completed the quantitative component of the survey (63 % completion rate). Participant flow through the study and reasons for declining to participate are illustrated in Fig. 1. Participants had a mean age of 63 years (±8.8 years; range 50–88), with the majority being female (59 %). A subset of these (n = 54; 16 %) partook in the semi-structured interview. The demographic characteristics of the interview sample were representative of the entire cohort. Table 1 describes the participants’ characteristics, medical conditions, and number of medications consumed daily, stratified by the CWC topic they selected.

**Table 1:** Respondent characteristics

Survey	Routine use of ECG (n = 105)	Antipsychotic use in dementia (n = 97)	Antibiotic use to treat sinusitis (n = 50)	Imaging for low back pain (n = 52)	Sedative-hypnotic use in the elderly (n = 40)	Overall (n = 344)
*Average age*						
Mean ± SD (years)	64.9 ± 9.9	62.9 ± 8.1	61.1 ± 7.0	62.2 ± 8.4	61.8 ± 9.0	63.0 ± 8.8
Range (years)	50–88	50–87	50–80	50–84	50–81	50–88
Female (%)	53.3	64.0	56.0	55.0	65.0	59.0
*Marital status* (*%*)						
Married	54.3	60.8	60.0	50.0	55.0	56.4
Single/unmarried	20.0	12.4	16.0	23.1	15.0	17.2
Divorced/separated	13.3	17.5	12.0	17.3	10.0	14.5
Widowed	10.5	8.2	10.0	7.7	10.0	9.3
*Place of birth* (*%*)						
Canada	59.0	66.0	70.0	69.2	77.5	66.3
USA	6.0	3.0	2.0	5.8	0.0	3.8
Other	33.0	28.0	26.0	23.1	22.5	27.9
*Living arrangements* (*%*)						
Lives alone	31.4	28.9	26.0	46.2	27.5	31.7
*Level of education* (*%*)						
Primary school	1.0	3.1	0.0	3.8	0.0	1.7
High school	9.5	20.6	20.0	23.1	7.5	16.0
College/University	87.6	75.3	76.0	67.3	92.5	79.9
Prefer not to disclose	1.9	1.0	4.0	5.8	0.0	2.3
*Health self-assessment* (*%*)						
Good to excellent	81.0	81.5	82.0	80.7	82.5	81.4
*Medical condition* (*%*)						
High blood pressure	53.3	34.0	30.0	32.7	27.5	38.4
Diabetes	21.1	16.5	14.0	13.5	7.5	16.0
Heart disease	22.9	9.3	12.0	1.9	7.5	12.2
Colon cancer	2.9	1.0	2.0	1.9	0.0	1.5
Asthma	9.5	10.3	18.0	13.5	2.5	10.8
Depression	16.2	20.6	14.0	9.6	30	17.7
*Number of different medications per day* (*%*)						
0	14.0	20.0	18.0	15.4	7.5	16.0
1–4	54.0	52.0	54.0	63.5	62.5	56.4
5–9	23.0	17.0	24.0	13.5	20.0	19.8
>10	9.0	7.0	4.0	5.8	10.0	7.2

### Choice of CWC topic

The most prevalent justification why participants selected a specific brochure topic was having a family member or friend impacted by the content addressed in the brochure (57 %). Other frequent themes were: no interest in the other brochures (37 %), applicability to the participant’s own health (35 %), or an interest in the content of the brochure (19 %).

### Baseline knowledge about CWC low-value interventions

At baseline, the proportion of respondents who correctly endorsed statements about low-value care ranged from 15.2 to 62.5 % (Table 2). Participants had the most accurate baseline knowledge related to the use of sedative-hypnotics (45–63 %; Table 2). Participants demonstrated the least accurate baseline knowledge for the use of antipsychotics (9–28 %; Table 2). The only predictor of a more accurate baseline perception for antipsychotic use was a higher educational level; older age (60+) was the only demographic variable statistically associated with accurate baseline perceptions for low back pain imaging. Baseline perceptions were not significantly associated with any other demographic variables for any of the other CWC topics.

**Table 2:** CWC knowledge

CWC Topic	Pre-endorsement of correct answer (%)	Post-endorsement of correct answer (%)	Proportion with knowledge improvement (95 % CI)	*P* value for change to the correct answer
*ECG educational brochure (n* *=* *105)*				
Routine ECGs are the best way to detect heart disease	15.2	41.9	30.5 (22.5–39.8)	<0.001
You should have an ECG done if you have diabetes	36.2	57.1	25.7 (18.3–34.8)	<0.001
Either question			47.6 (38.3–57.1)	
*Antipsychotics educational brochure (n* *=* *97)*				
Antipsychotic medication is the best available option to treat disruptive behaviour in people with dementia	27.8	83.5	57.7 (47.8–67.1)	<0.001
Health Canada has not approved the use of antipsychotic medications to treat disruptive behaviour in people with dementia	9.3	59.8	51.5 (41.7–61.2)	<0.001
Either question			74.2 (64.7–81.9)	
*Sinusitis educational brochure (n* *=* *50)*				
One in four people who take antibiotics have side effects	58.0	86.0	34 (22.4–47.8)	0.002
Antibiotics are not necessary to treat sinus infections	44.0	84.0	46 (33–59.6)	<0.001
Either question			66 (52.2–77.6)	
*Back pain educational brochure (n* *=* *52)*				
An imaging test taken within the first week of having lower back pain can help you heal faster	26.9	71.2	44.2 (31.6–57.7)	<0.001
Imaging tests for lower back pain have no risks	50.0	80.8	36.5 (24.8–50.1)	0.001
Either question			59.6 (46.1–71.8)	
*Sleeping pills educational brochure (n* *=* *40)*				
Sleeping pills are mild tranquilizers that are safe when taken for long periods of time	45.0	72.5	35 (22.1–50.5)	0.008
Sleeping pills cause no side effects	62.5	85.0	27.5 (16.1–42.8)	0.013
Either question			40 (26.3–55.4)	

### Improvement in knowledge

Post-intervention, the proportion of respondents endorsing correct answers about low-value care ranged from 41.9 to 85 % (Table 2). Knowledge improved significantly (p < 0.05) for all CWC topics. The proportion of respondents whose knowledge improved for each CWC topic is illustrated in Table 2. No demographic factors were associated with knowledge improvement.

### Intent to discuss low value care

After reading any of the CWC patient educational materials, 70 % of patients intended to discuss the content of the educational material with a health care professional, family member, or friend (Table 3). Forty-eight percent specifically said that they would discuss the material with a health care provider. Across all five topics, patients who intended to discuss low-value care exhibited significant knowledge improvement on the pre-post questionnaire (p = 0.004).

**Table 3:** Intention to discuss CWC topics and intent to change behaviour

	% that do not intend to discuss (95 % CI)	% that intend to discuss with health professionals^a^ (95 % CI)	% that intend to discuss with family, relatives and friends (95 % CI)	% will change behaviour (95 % CI)
ECG educational brochure (n = 105)	29.5 (21.6–38.8)	52.4 (42.9–61.7)	32.4 (24.2–41.8)	36.2 (27.6–45.7)
Antipsychotics educational brochure (n = 97)	27.8 (19.9–37.5)	37.1 (28.2–47)	53.6 (43.7–63.2)	61.9 (51.9–70.9)
Sinusitis educational brochure (n = 50)	30.0 (19.1–43.8)	46.0 (33–59.6)	40.0 (27.6–53.8)	42.0 (29.4–55.8)
Back pain educational brochure (n = 52)	23.1 (13.7–36.1)	61.5 (48–73.5)	30.8 (19.9–44.3)	48.1 (35.1–61.3)
Sleeping pills educational brochure (n = 40)	40.0 (26.3–55.4)	45.0 (30.7–60.2)	35.0 (22.1–50.5)	30 (18.1–45.4)
Overall (n = 344)	29.4 (24.8–34.4)	47.7 (42.5–52.9)	39.5 (34.5–44.8)	45.3 (40.2–50.6)

*CI* confidence intervals

^a^ Healthcare professional = physician, nurse, pharmacist and other healthcare professionals

### Intention to change behaviour

The overall proportion of patients that stated an intention to incorporate the brochure’s recommendations into their future health behaviours was 45 % (Table 3). The majority of participants (55 %) who indicated that the brochure’s contents would not change their health behaviours answered this way because they already incorporated the health behaviours that the brochures advocated (69 %). Other reasons why participants did not anticipate changing their behaviour and selected quotes are found in Table 4. Forty-five out of the 54 (83.3 %) participants who partook in the interviews already espoused the views on unnecessary care described in the brochure, or admitted readiness to adopt these views in the future.

**Table 4:** Reasons why CWC education may or may not change primary care patient behaviour

Will CWC information change your behaviour?	
If yes, how?	If not, why not?
Will take action	I already hold the views of CWC
“I will ask my friends if they are taking antipsychotic drugs” “I will certainly ask—I don’t think she’s in medications but I will verify that. I now will be more savvy when advocating for her” “We will exercise and eat better and when the doctor says they will give a test, I will ask if it is necessary and whether it has to be done annually” “I would certainly have conversations with family members and caregivers and want to know what they are taking” “I would discourage antipsychotics for dementia, unless other things have not helped” “I will talk to my friends about antipsychotic use and if they are concerned, I will educate them”	“Not really. I try to be careful anyways and so I am already doing the things you are suggesting” “I self monitor and I am an RN so I know what CWC is telling me and my views conform with what CWC says; I listen to doctors and would question if I didn’t agree with the recommendation” “I question physician recommendations anyways and my views conform with Choosing Wisely. I always discuss why I need test, procedures, and exams” “I have already been exposed to this information and I already agree with this” “I already believe that overtesting is a waste of money, can cause more problems with more false positives and harms”
Will ask more questions	Trust physician judgment
“I will question physician decisions more and I will be more assertive. I will take more responsibility for asking questions. I need a good reason to take medications and I will take more control over my health” “Now that I am aware of this, if a professional recommended an antipsychotic, I might inquire more about it than I would have otherwise; I’d ask if there were other courses if action and if it was really necessary. I’d now wonder whereas before I wouldn’t have” “The next time a doctor recommends a certain test, I will do more research, ask questions and not feel obligated because a health care practitioner recommends it” “I would question more especially for tests and medications”	“It wouldn’t change my behaviour—because I trust what the doctor says” “I trust my doctor to tell me which tests to have” “I have a great doctor that doesn’t send me for anything unnecessary; I trust him unreservedly and so because I already have that care, I don’t need to change my behaviour”
	I should have as many tests as possible to be safe
	“Prevention is important. My father had an ECG and it saved his life so I think getting routine preventive tests all the time, even if not necessary, is important” “It will not—because my sister died, I now get tests for everything”

## Discussion

Previous studies have queried primary care physicians and other medical specialists about their awareness of Choosing Wisely [16, 17]; however this is the first patient survey in primary care to ascertain whether patients are aware of and ready to engage in de-adoption of low-value interventions. Almost 85 % of participants already espoused or were ready to adopt the philosophy of Choosing Wisely. After reading the CWC brochures, the majority intended to discuss the information with a health care provider, friend or family member. Our study provides evidence that distribution of CWC patient educational materials in the waiting rooms of ambulatory clinics can improve patient knowledge around the use of unnecessary care, regardless of age, sex or educational status. As expected, acquisition of new knowledge was most striking for topics with poor baseline understanding. For instance, only 28 % of participants correctly endorsed the statement “antipsychotics are not the best treatment for disruptive behaviour” prior to reading the brochure, whereas 83 % agreed afterwards.

Despite a growing interest in de-investment in low-value practices internationally [15, 21], the optimal approach to de-adopting these practices in primary care remains unknown. What is becoming clear is that patients have an important role to play in the de-adoption process. Many older adults already adhere to the Choosing Wisely philosophy or can be easily convinced of its merits [8, 11]. A number of methods exist to enlist patients in decisions about reducing low value interventions in primary care, the most obvious being direct communication with patients during the clinician-patient visit. However, not all physicians are skilled in patient-centred communication, and others express concern that effective communication takes time, something that is in short supply with the pressure to see more patients in a day [22].

Delivery of information to patients through printed or online educational material is another way to potentially accelerate the reduction of low-value care. Almost half of the patients exposed to the CWC materials in our study voiced readiness to change behaviour. Although intent does not always lead to action [17, 23], 27 % of patients in the EMPOWER trial succeeded in discontinuing their benzodiazepines at 6-months after receiving a mailed educational brochure on the comparative benefits versus harms of these medications [17]. Those who achieved discontinuation represented a substantial portion of the individuals that originally expressed an intent to discontinue treatment.

The qualitative findings from our study (Table 4) suggest that de-prescription of medication may prove easier than the reduction of low-value screening services. Patients who most commonly expressed reluctance to change behaviour were those who felt strongly about obtaining regular screening tests. A recent clinical trial examined the willingness of primary care patients aged 50–85 to reduce low-value screening services, after exposure to various forms of written educational material [24]. There was no change in intent for screening after exposure to one of four different decision aids aimed at discouraging patients from participating in colorectal cancer screening, osteoporosis screening or prostate cancer screening in low-risk individuals. Efforts to reduce low-value care may therefore require different approaches, depending on the topic.

Although the results of our survey are promising for the distribution of CWC educational materials to patients in the waiting rooms of primary care practices, some caveats apply. Our sample was highly educated, likely due to selection bias towards individuals who had heard of Choosing Wisely Canada, and who chose to participate in the study. We therefore cannot generalize our results across all educational strata. Furthermore, patients were tested on their knowledge of the statements in the CWC brochures; we did not use a formal validated measure for assessing the overuse of low-value interventions. In addition, our study findings may not be applicable outside of Canada and to other patient groups. The findings may have been influenced by social desirability bias and selection bias, owing to the fact that there was no control or comparison group. Finally, we only selected five different CWC topics. We do not know if patients’ readiness to de-adopt other low-value interventions would be as high as for the ones tested in this study.

## Conclusions

For the most part, older primary care patients express a strong readiness to de-adopt annual electrocardiogram screening, the use of antipsychotic drugs for patients with dementia, the use of antibiotics for treatment of sinusitis, imaging for low back pain, and the use of sedative-hypnotic drugs for insomnia. CWC brochures effectively improved patient knowledge in these five topic areas, when distributed in the waiting rooms of primary care practices. Equipping patients with information may be an appropriate first step towards facilitating potentially challenging conversations around unnecessary care. Future studies are needed to determine if the distribution of CWC patient educational materials in primary care clinics leads to a measurable reduction in unnecessary tests and treatments.

## Abbreviations

CWC: Choosing Wisely Canada
ECG: electrocardiogram testing
